# Extended preconditioning on soft matrices directs human mesenchymal stem cell fate via YAP transcriptional activity and chromatin organization

**DOI:** 10.1063/5.0124424

**Published:** 2023-02-22

**Authors:** Yufei Ma, Xu Zhang, Shaoxin Tang, Li Xue, Jing Wang, Xiaohui Zhang

**Affiliations:** 1 Bioinspired Engineering and Biomechanics Center (BEBC), The Key Laboratory of Biomedical Information Engineering of Ministry of Education, Xi'an Jiaotong University, Xi'an 710049, People's Republic of China; 2 The Key Laboratory of Biomedical Information Engineering of Ministry of Education, Center for Mitochondrial Biology and Medicine, School of Life Science and Technology, International Joint Laboratory for Micro/Nano Manufacturing and Measurement Technology, Xi'an Key Laboratory for Biomedical Testing and High-End Equipment, Xi'an Jiaotong University, Xi'an 710049, People's Republic of China

## Abstract

Dynamic extracellular matrix (ECM) mechanics plays a crucial role in tissue development and disease progression through regulation of stem cell behavior, differentiation, and fate determination. Periodontitis is a typical case characterized by decreased ECM stiffness within diseased periodontal tissues as well as with irreversible loss of osteogenesis capacity of periodontal tissue-derived human periodontal tissue-derived MSCs (hMSCs) even returning back to a physiological mechanical microenvironment. We hypothesized that the hMSCs extendedly residing in the soft ECM of diseased periodontal tissues may memorize the mechanical information and have further effect on ultimate cell fate besides the current mechanical microenvironment. Using a soft priming and subsequent stiff culture system based on collagen-modified polydimethylsiloxane substrates, we were able to discover that extended preconditioning on soft matrices (e.g., 7 days of exposure) led to approximately one-third decrease in cell spreading, two-third decrease in osteogenic markers (e.g., RUNX2 and OPN) of hMSCs, and one-thirteenth decrease in the production of mineralized nodules. The significant loss of osteogenic ability may attribute to the long-term residing of hMSCs in diseased periodontal tissue featured with reduced stiffness. This is associated with the regulation of transcriptional activity through alterations of subcellular localization of yes-associated protein and nuclear feature-mediated chromatin organization. Collectively, we reconstructed phenomena of irreversible loss of hMSC osteogenesis capacity in diseased periodontal tissues in our system and revealed the critical effect of preconditioning duration on soft matrices as well as the potential mechanisms in determining ultimate hMSC fate.

## I. INTRODUCTION

Mesenchymal stem cells (MSCs) have demonstrated great potential in cell therapeutics and regenerative medicine due to their multipotency.^1,2^ The differentiation of MSCs can be regulated not only by biochemical cues (e.g., cytokines, growth factors, and small bioactive molecules^3–5^) but also by biophysical cues from the surrounding cell microenvironment (e.g., surface topography, matrix stiffness, pore size, and external mechanical forces^6–9^). In the past decades, intensive research attention has been paid to exploring the regulating effect and the underlying mechanism of biophysical cues, especially the extracellular matrix (ECM) mechanics, on stem cell behavior and lineage specification.^10–12^ The stem cells was revealed to possess mechanosensory machinery, through which they can sense and respond to the mechanical stimuli by converting biophysical cues into biochemical signals.^13,14^ Up to now, a number of mechanotransduction pathways linking the mechanical microenvironment to the nucleus have been identified. Examples range from the direct activation of ion channels,^15,16^ the interactions among integrin, cytoskeleton, and nucleoskeleton,^17–19^ the translocation of transcriptional coactivator, Yes-associated protein (YAP) between the cytoplasm and the nucleus,^20–22^ to the chromatin remodeling mediated alteration in transcriptional activity,^23,24^ all playing crucial parts in stem cell lineage commitment. Cumulative understanding of the mechanotransduction mechanisms enables us to precisely control the stem cell fate through the manipulation of ECM mechanics.

The effects of ECM mechanics on MSC differentiation have been extensively studied, while most work employed static mechanical systems. In fact, the ECMs in the living systems are highly dynamic, constantly undergoing temporal and spatial remodeling. Therefore, the ECMs exhibit varying biomechanical characteristics throughout the lifespan, which has proven to be critical for tissue development or disease progression (e.g., fibrosis, cancer).^25,26^ As a result, a range of platforms with the ability to provide dynamic stiffness cues have been developed and utilized to explore the effect of temporal stiffening or softening on modulating cell behavior and fate determination. For instance, the cardiomyocytes experience ECM stiffening up to ninefold from mesoderm to maturation, which has proven to be essential for heart development.^27^ To recapitulate this temporal stiffening process, a thiolated-hyaluronic acid hydrogel system with the ability to adjust stiffness by changing the molecular weight of crosslinkers was developed.^27^ The pre-cardiac cells cultured on the stiffening hydrogels exhibited a threefold increase in the expression of mature cardiac specific markers and formed 60% more maturing muscle fibers compared to the static hydrogel groups. With this system, the importance of ECM stiffening in cardiomyocyte differentiation and maturation was revealed. Fibrosis is another typical case, featured by tissue hardening with excessive ECM accumulation in and around the inflamed/damaged tissues. To investigate the effect of fibrosis regression on the cells in liver, a dynamic softening ECM based on methacrylated hyaluronic acid (MeHA) hydrogels was constructed.^28^ The softening of matrices (∼20 to ∼3 kPa) was able to induce the phenotypic of hepatic stellate cells from myofibroblast to fibroblast, suggesting the crucial effect of ECM mechanics on fibrosis regression.

Moreover, cells have exhibited the ability to remember and continue processing previous mechanical information even after being transferred to current mechanical microenvironment and even lead to a great influence on ultimate cell fate.^20,29,30^ Anseth group once demonstrated that with sufficient long exposure to stiff substrates, hMSCs could still remember the past mechanical cues when transferred to relatively soft substrates and retain their osteogenic differentiation with irreversible Yes-associated protein (YAP) nuclear localization.^20^ They further discovered that extended exposure to stiff substrates can result in epigenetic modifications and persistent chromatin remodeling, which played a critical role in the storage of previously experienced mechanical cues and ultimate cell fate determination.^29^ A similar phenomena was observed in another study, where microRNA-21 was identified as a key player of fibrogenic mechanical memory of MSCs.^31^ By regulation through the mechanosensitive myocardin-related transcription factor-A (MRTF-A), the microRNA-21 levels of MSCs on the stiff substrates gradually increased and remained high even after removing the mechanical stimulus. While these studies elaborate how the mechanical memory induced by pre-culture on stiff substrates can influence ultimate cell fate, however, whether MSCs can similarly store the preconditioned soft mechanical information, or such mechanical memory can further affect the ultimate cell fate after being transferred to relatively stiff substrates have not yet been studied.

Periodontitis, as an infectious oral disease, can damage the supporting periodontal tissues and the alveolar bone supporting the teeth.^32^ The diseased periodontal tissue is commonly accompanied by decreased ECM stiffness and MSCs losing osteogenic ability even after being transferred to physiological cellular microenvironment. We hypothesize that the irreversible loss of MSC osteogenic capacity could be due to the long-term residing in the diseased periodontal tissue with reduced ECM stiffness. In this work, we developed a mechanical culture system by using collagen-modified polydimethylsiloxane (PDMS) matrices. The human periodontal tissue-derived MSCs (hMSCs) initially cultured on soft substrates with either short- or long-term preconditioning were later transferred to stiff substrates to identify the effect of soft priming and duration on ultimate cell fates. The potential mechanisms leading to the weakened osteogenic ability of hMSCs induced by long-term preconditioning on soft substrates was further revealed, particularly identifying the effect of subcellular localization of YAP and nuclear feature-mediated chromatin organization on the regulation of transcriptional activity. This study may provide new insight into how preconditioning on soft matrices affects ultimate hMSC fate and offer explanation for the phenomena observed for hMSCs in the diseased periodontal tissues.

## II. RESULTS AND DISCUSSION

### A. Fabrication and characterization of stiff and soft matrices

To fabricate stiff and soft matrices, we prepared collagen-coated polydimethylsiloxane (PDMS) substrates with high and low modulus, respectively. By changing the mass ratio of base to curing agent, PDMS substrates with compressive moduli of approximate 60 and 26 kPa were fabricated, respectively (Fig. S1, supplementary material). Considering the intrinsic hydrophobicity of the PDMS substrates, the PDMS substrates were incubated in a collagen type I solution to improve cell adhesion.^33^ Upon surface modification, similar topological microstructures were observed for PDMS substrates with both high and low modulus (Fig. S2, supplementary material). Meanwhile, the surface modification did not result in any significant difference in compressive moduli (Fig. S1, supplementary material). These results indicated the successful fabrication of stiff and soft matrices by using collagen-coated PDMS substrates, which might possibly be utilized to represent the stiffness of ECM of healthy and diseased periodontal tissues *in vitro*, respectively.

**FIG. 1.**
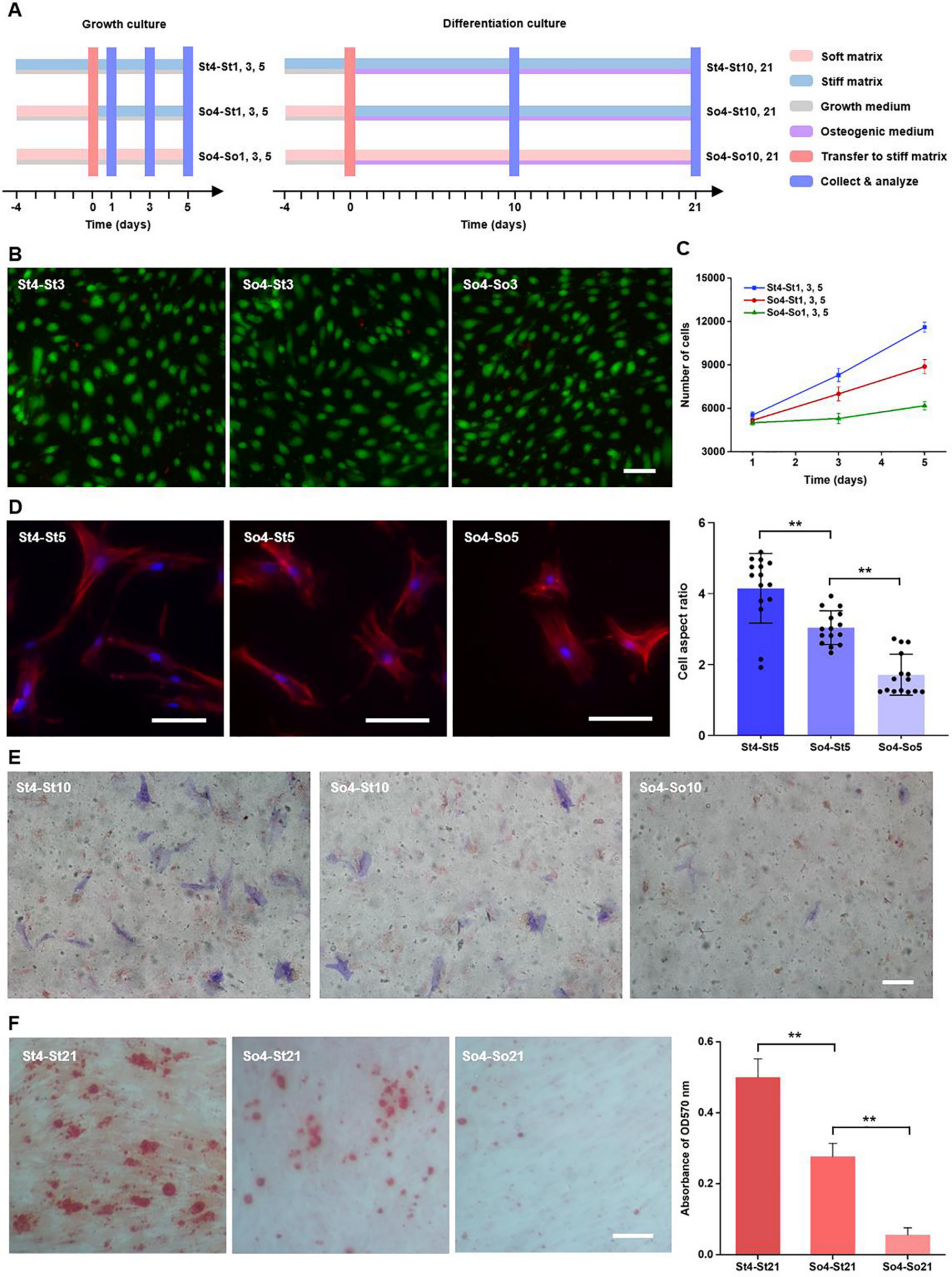
Characterizations of proliferation, morphology, and differentiation of hMSCs preconditioned on soft matrices. (a) Schematic illustration of preconditioning of hMSCs on soft matrices. For growth culture, hMSCs were first cultured on soft matrices in growth media for 4 days and then transferred to stiff matrices and cultured subsequently in growth media for 1, 3, and 5 days before analysis. For differentiation culture, hMSCs were first cultured on soft matrices in growth media for 4 days and then transferred to stiff matrices and cultured subsequently in osteogenic media for 10 and 21 days before analysis. hMSCs initially cultured on stiff or soft matrices, and transferred to the same matrices and cultured subsequently under the same condition were set as controls. (b) Live/dead staining confocal microscopy images of hMSCs 3 days after transfer. Scale bar: 100* μ*m. (c) Cell proliferation was analyzed by CCK-8 assay after transferring to a new matrix. n = 3 (with biological replicates). (d) Phalloidin-staining images showing cell morphology and corresponding cell aspect ratios 5 days after transfer (n = 15 cells from biological triplicate). Scale bar: 100 *μ*m. ^**^p < 0.01. (e) ALP staining of hMSCs cultured in osteogenic media 10 days after transfer. Scale bar: 100 *μ*m. (f) Alizarin red S staining images of hMSCs cultured in osteogenic media and quantitative retention analysis 21 days after transfer. n = 3 (with biological replicates). Scale bar: 100 *μ*m. ^**^p < 0.01.

**FIG. 2.**
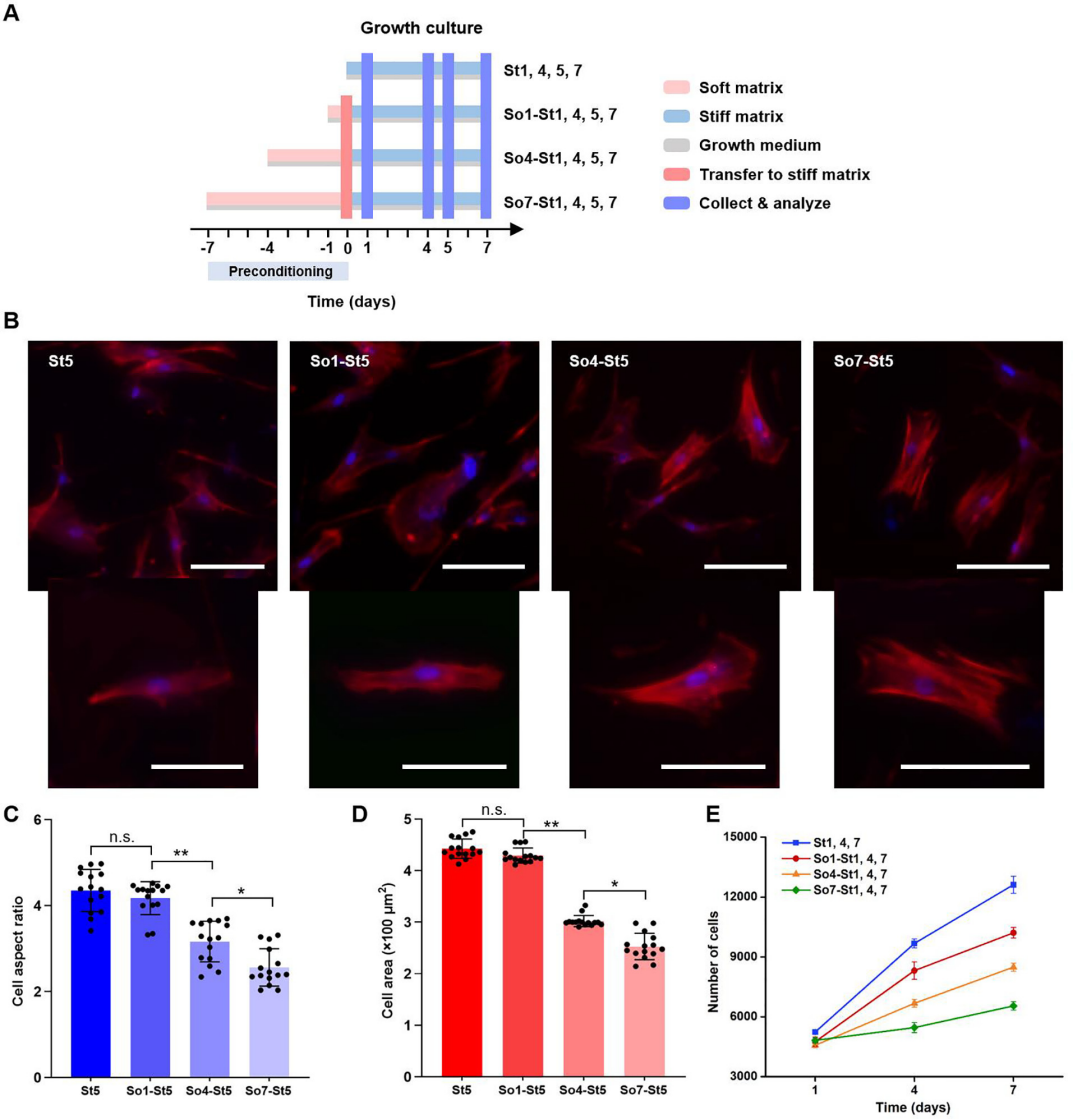
Extended preconditioning on soft matrices changes cell morphology, spreading, and proliferation of hMSCs. (a) Schematics illustrating the growth culture of hMSCs with preconditioning on soft matrices. Cells were initially cultured on soft matrices in growth media for 1, 4, and 7 days, respectively, and then transferred to stiff matrices and cultured in growth media for 1, 4, 5, and 7 days, respectively, before analysis. The cells cultured on stiff matrices without mechanical preconditioning were set as controls. (b) Representative confocal microscopy images of cell morphology on day 5 after cell transfer to stiff matrices. Scale bar: 100 *μ*m. Quantitative analysis (n = 15 cells from biological triplicate) of (c) cell aspect ratio and (d) cell area of hMSCs cultured under indicated conditions. ^*^p < 0.05, ^**^p < 0.01. (e) Cell proliferation on days 1, 3, and 5 after transferring to stiff matrices by using CCK-8 assay. n = 3 (with biological replicates).

### B. Preconditioning of hMSCs on soft matrices affects cell behavior and fate determination

To investigate the effect of soft priming on hMSC behaviors, the cells were seeded on the soft matrices for 4 days, and then transferred to stiff matrices and cultured for 1, 3, and 5 days, respectively, before analysis (i.e., So4-St1, 3, and 5). The cells initially cultured on stiff or soft matrices for 4 days, then cultured on stiff or soft matrices for 1, 3, and 5 days, respectively, were prepared as controls (i.e., St4-St1, 3, and 5 and So4-So1, 3, and 5) [Fig. 1(a)]. The hMSCs exhibited high viability (>95%) in all the study groups with no significant difference [Figs. 1(b) and S3, supplementary material]. However, there is significant difference of hMSC proliferation among different groups [Fig. 1(c)]. Specifically, the hMSCs in the So4-St1, 3, and 5 group showed significantly higher proliferation rate compared to that in the So4-So1, 3, and 5 group, but significantly lower proliferation rate than that in the St4-St1, 3, and 5 group. As shown in the confocal microscopy images, the cells in the St4-St5 group exhibited typical elongated morphology with larger spreading areas, while those in the So4-St5 and So4-So5 groups had much smaller spreading areas. The quantitatively analysis indicated that the cell aspect ratio of hMSCs was significantly decreased in the So4-St5 group compared to that in the St4-St5 group and was further reduced in the So4-So5 group [Fig. 1(d)]. These results suggest that preconditioning on soft matrices has adverse effect on cell spreading and elongation and thus proliferation.

We further assessed the influence of preconditioning on soft matrices on the osteogenic differentiation of hMSCs. As illustrated in Fig. 1(a), the hMSCs were pre-cultured on soft matrices in growth media for 4 days and then transferred to stiff matrices and cultured in osteogenic media for 10 and 21 days, respectively, before analysis (i.e., So4-St10, 21). Correspondingly, the cells cultured and transferred to the same matrices (stiff or soft) with the same culture procedures as above were set as control groups (i.e., St4-St10, 21 and So4-So10, 21). Alkaline phosphatase (ALP) activity, a marker of osteogenesis, was used to evaluate the osteogenic differentiation of hMSCs on different matrices. The expression level of ALP in the So4-St10 group was significantly lower than that in the St4-St10 group [Figs. 1(e) and S4, supplementary material]. This trend was further confirmed with the Alizarin red S staining results, an indicator for late osteogenic differentiation based on the formation of mineralized nodules.^34^ The orange-red precipitates obviously reduced in the So4-St21 group relative to the St4-St21 group [Fig. 1(f)]. These results together suggested that the preconditioning on soft matrices significantly hindered the osteogenesis of hMSCs. In previous works, MSCs cultured on ∼26 kPa substrates (regarded as stiff matrices sometimes) exhibited a certain amount of osteogenesis capacity.^35,36^ In the current study, hMSCs preconditioned and subsequently cultured on soft matrices with stiffness of ∼26 kPa (e.g., So4-So10 and So4-So21) showed a little bit weaker osteogenesis capacity compared to previous studies, which was probably due to intrinsically limited osteogenesis capacity of periodontal tissue-derived MSCs.

### C. Extended preconditioning of hMSCs on soft matrices regulates cell behavior and fate determination

The effect of soft priming duration on cell behaviors, including cell morphology, spreading, and proliferation, was further examined. The hMSCs were initially cultured on soft matrices supplemented with growth media for 1, 4, and 7 days, and then transferred to stiff matrices and cultured for 1, 4, 5, and 7 days, respectively, before analysis (i.e., So1-, So4-, and So7-St1, 4, 5, 7). Cells cultured on stiff matrices without preconditioning on soft matrices were set as controls (i.e., St1, 4, 5, and 7) [Fig. 2(a)]. The cells continuously cultured on stiff matrices exhibited typical spindle morphology with more spreading (e.g., St5), and no significant change was observed when preconditioning on soft matrices for 1 day (e.g., So1-St5). However, with extended time of preconditioning (4 and 7 days), the cells exhibited much less spreading morphology. This was further confirmed with the quantitative analysis of cell aspect ratio and spreading area [Figs. 2(b)–2(d)]. In addition, cell proliferation was also closely related to the preconditioning duration on soft matrices. As present in Fig. 2(e), the proliferation of hMSCs was significantly inhibited with an increase in extended preconditioning on soft matrices, with a half-fold decrease in So7-St7 group.

**FIG. 3.**
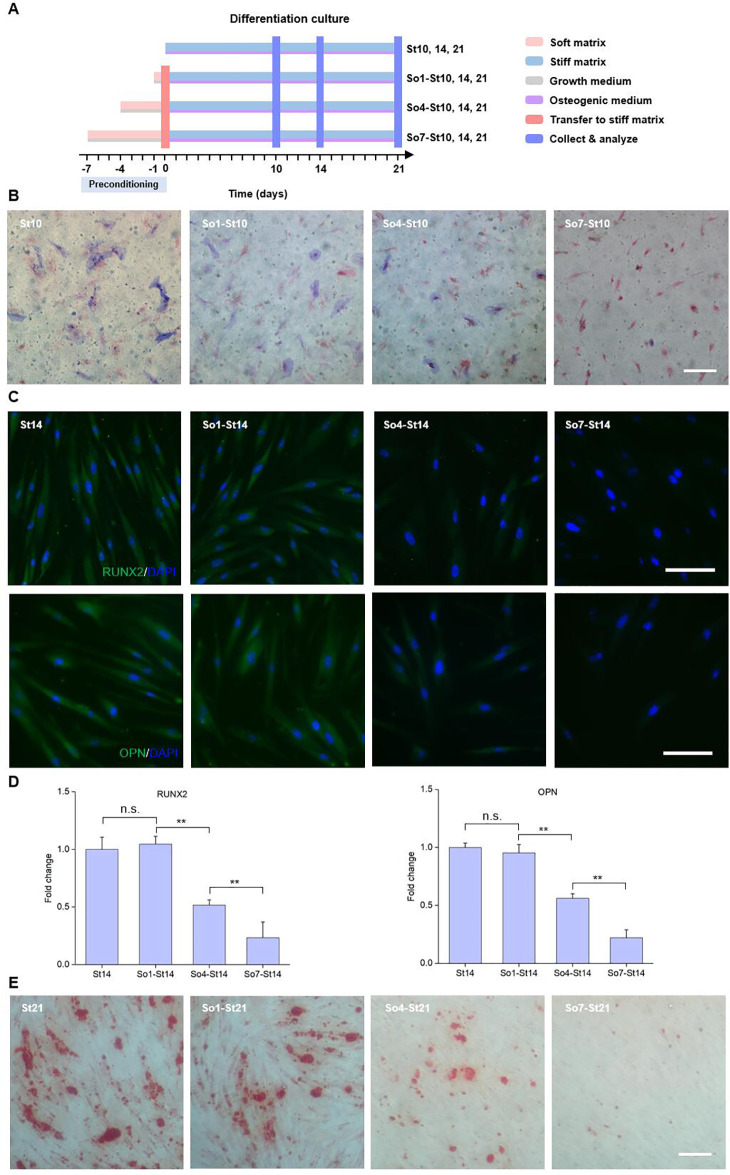
Extended preconditioning on soft matrices affects osteogenic differentiation of hMSCs. (A) Schematics illustrating the differentiation culture of hMSCs with preconditioning on soft matrices. Cells were cultured on soft matrices in growth media for 1, 4, and 7 days, respectively, before trypsinization, and then transferred to stiff matrices and cultured in osteogenic media for 10, 14, and 21 days, respectively, before collection and analysis. The cells cultured on stiff matrices without mechanical preconditioning were as controls. (b) ALP staining of the hMSCs cultured on stiff matrices in osteogenic media on day 10 after preconditioning on soft matrices. Scale bar: 100 *μ*m. (C) Representative confocal microscopy images of RUNX2 and OPN expression in hMSCs cultured on stiff matrices in osteogenic media on day 14 after preconditioning on soft matrices. Scale bar: 100 *μ*m. (d) mRNA expression levels of RUNX2 and OPN in hMSCs on day 14 with varied preconditioning times on soft matrices. Fold change is expressed in relative to the St14. n = 3 (with biological replicates), ^**^p < 0.01. (e) Alizarin red staining of the hMSCs cultured on stiff matrices in osteogenic media on day 21 after preconditioning on soft matrices. Scale bar: 100 μm.

We further studied the effect of preconditioning duration on soft matrices on the osteogenic differentiation of hMSCs. For this purpose, the cells were cultured in osteogenic media rather than growth media after transferred to stiff matrices (i.e., So1-, So4-, and So7-St10, 14, 21). Cells cultured on stiff matrices in osteogenic media without preconditioning were set as controls (i.e., St10, 14, and 21) [Fig. 3(a)]. The cells cultured continuously on stiff matrices (e.g., St10) or with 1 or 4 days of mechanical preconditioning (e.g., So1-St10 and So4-St10) exhibited a certain amount of ALP expression. However, a reduction in ALP expression was observed for the cells experiencing long-term mechanical preconditioning (e.g., So7-St10) [Fig. 3(b) and S5, supplementary material). We further looked into the expression of osteogenesis specific proteins RUNX2 and OPN. Compared to the control, preconditioning on soft matrices for 1 day did not result in significant changes in RUNX2 and OPN expressions. However, extension in the preconditioning duration to 4 or 7 days significantly decreased the RUNX2 expression in cell nucleus and OPN expression [Figs. 3(c) and 3(d) and S6 and S7, supplementary material]. Similar trends were observed for the production of mineralized nodules in hMSCs with different preconditioning durations. The decline in alizarin red retention was observed along the increase in extended soft priming duration, with a lowest production of mineralized nodules in So7-St21 group [Figs. 3(e) and S8, supplementary material). These results indicate that the extended preconditioning duration on soft matrices could effectively regulate cell behavior and reduce osteogenic differentiation of hMSCs.

### D. Preconditioning duration on soft matrices affects YAP transcriptional activity

**FIG. 4.**
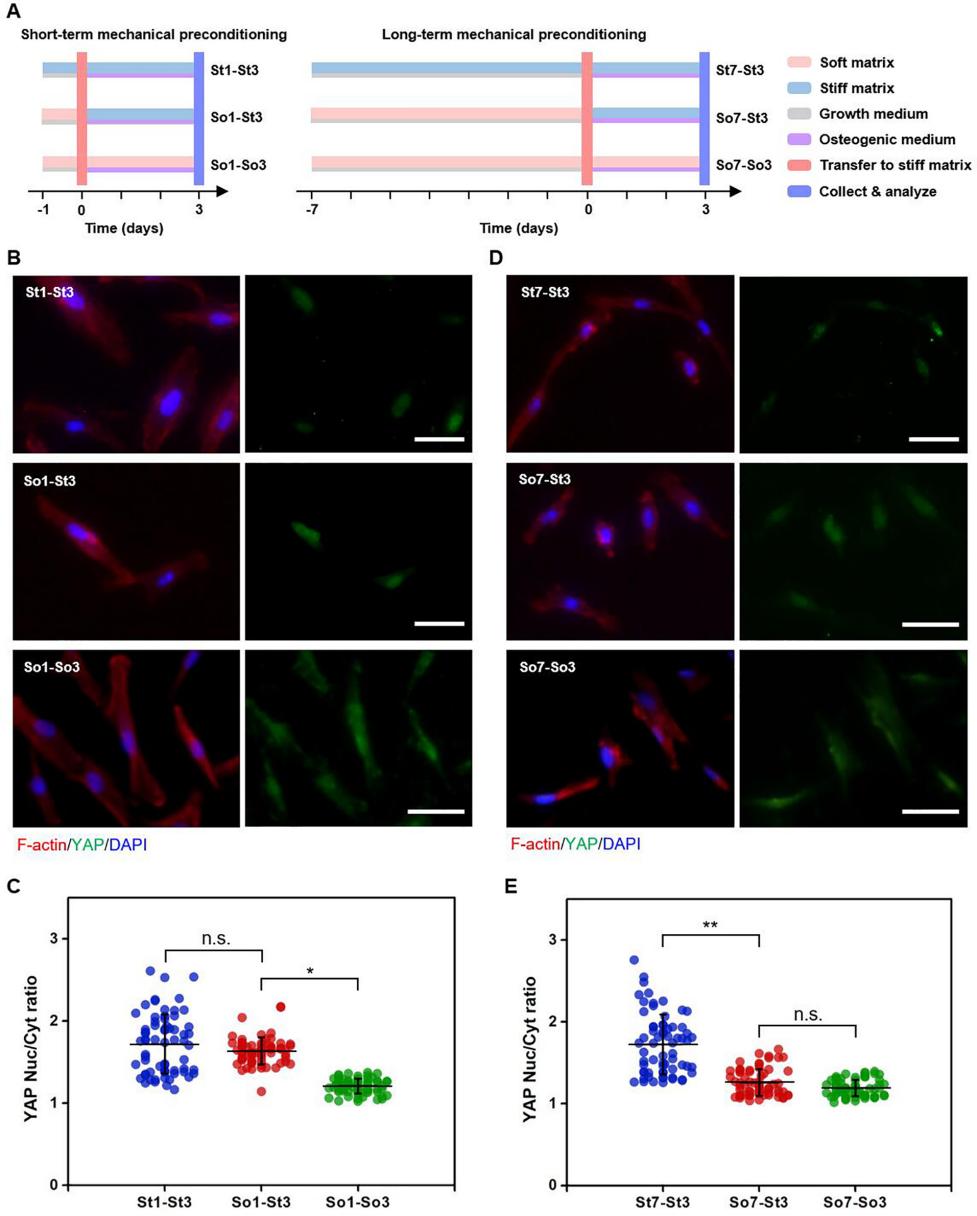
Localization of YAP after short-term and long-term preconditioning of hMSCs on soft matrices. (a) Schematic illustration of short-term and long-term mechanical preconditioning. hMSCs were cultured on soft matrices in growth media for 1 day (i.e., short-term mechanical preconditioning) or 7 days (i.e., long-term mechanical preconditioning) before trypsinization and then transferred to stiff matrices and cultured in osteogenic media for 3 days before collection and analysis. The cells initially cultured on stiff or soft matrices for 1 day or 7 days and then transferred to stiff or soft matrices and cultured for 3 days were set as controls. (b) Representative confocal microscopy images of YAP expression in hMSCs after short-term mechanical preconditioning, and (c) corresponding quantitation (n = 66 cells from biological triplicate) of YAP Nuc/Cyt ratio. ^*^p < 0.05. (d) Representative confocal microscopy images of YAP expression in hMSCs after long-term mechanical preconditioning, and (e) corresponding quantitation (n = 66 cells from biological triplicate) of YAP Nuc/Cyt ratio. ^**^p < 0.01.

To identify the underlying mechanism of extended soft priming-regulated hMSC behavior and fate, we examined the YAP localization in hMSCs under short-term and long-term mechanical preconditioning, respectively. The cells were initially cultured on soft matrices for 1 day (i.e., short-term mechanical preconditioning) or 7 days (i.e., long-term mechanical preconditioning) and then transferred to stiff matrices and cultured for 3 days before analysis (i.e., So1-St3 and So7-St3) [Fig. 4(a)]. Since nuclear localization is an indicator of active YAP, the subcellular localization of YAP was assessed using immunofluorescence images. As shown in Figs. 4(b)–4(e), hMSCs cultured on stiff matrices exhibited predominant YAP nuclear localization regardless of culture time (e.g., St1-St3 and St7-St3). In contrast, cells sequentially exposed to soft matrices displayed significant YAP cytoplasmic localization, indicating deactivation of YAP (e.g., So1-So3 and So7-So3), consistent with previous reported studies.^20,29^ The soft priming duration exhibited to be an important regulator for YAP activation/deactivation. After short-term preconditioning on soft matrices, YAP converted primarily to nuclear localization after the hMSCs were transferred to stiff matrices and cultured for 3 days, comparable to that in St1-St3 group [Fig. 4(b)]. Quantification of the immunofluorescence staining revealed no obvious difference in YAP Nuc/Cyt ratio between St1-St3 and So1-St3 groups, but a significant increase in YAP Nuc/Cyt ratio in cells in So1-St3 group compared to that in So1-So3 group [Fig. 4(c)], suggesting the incapability of short-term preconditioning on soft matrices hold the deactivation of YAP once transferred and cultured on stiff matrices.

After long-term preconditioning on soft matrices, YAP remained in the cytoplasm even after hMSCs were transferred to stiff matrices, indicating a deactivated state of YAP in hMSCs in So7-So3 group [Fig. 4(d)]. Quantification of the immunofluorescence staining demonstrated a significant decrease in YAP Nuc/Cyt ratio in hMSCs in So7-St3 group relative to that in St7-St3 group and a comparable YAP Nuc/Cyt ratio in So7-St3 to that in So7-So3 group [Fig. 4(e)]. This indicated the critical role of long-term preconditioning on soft matrices in maintaining YAP deactivation state even though the cells were subsequently transferred to stiff matrices. All these results suggested that extended preconditioning on soft matrices could regulate cell behavior and fate determination, to some extent, through the mediation of YAP localization, namely, its activity.

### E. Nuclear mechanics, nuclear volume and shape, and chromatin organization in hMSCs depend on the preconditioning duration on soft matrices

**FIG. 5.**
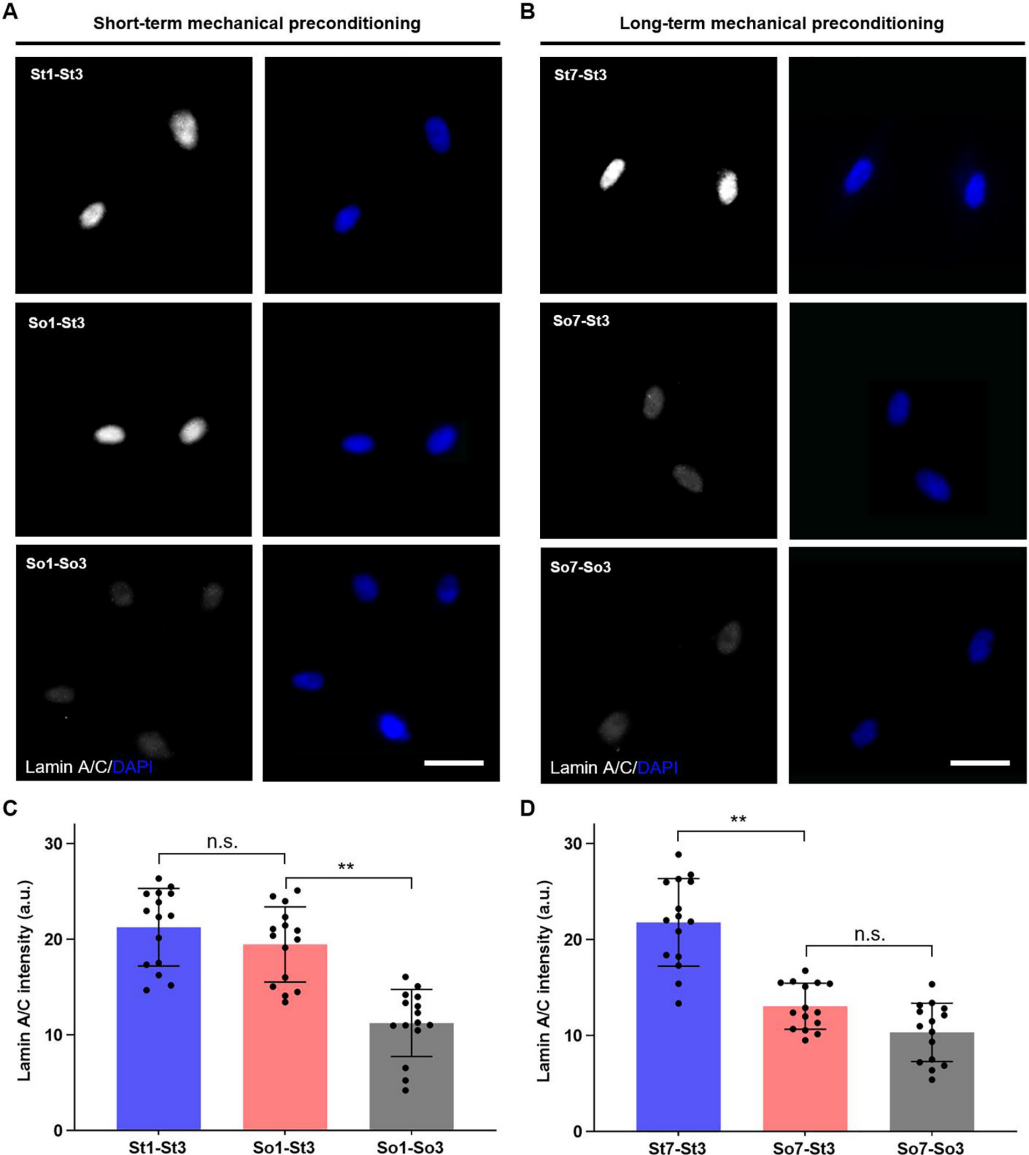
Influences of short-term and long-term mechanical preconditioning on nuclear mechanics of hMSCs. Representative confocal microscopy images of lamin A/C expression in hMSCs with (a) short-term and (b) long-term preconditioning on soft matrices. Scale bar: 50 μm. Corresponding quantitation (n = 15 cells from biological triplicate) of lamin A/C expression in hMSCs with (c) short-term and (d) long-term preconditioning on soft matrices. ^**^p < 0.01.

To further reveal the mechanism of mechanical preconditioning duration on directing cell fate of hMSCs, the time lines of short-term and long-term mechanical preconditioning [Fig. 4(a)] were subsequently employed to investigate nuclear mechanics of hMSCs. As shown in confocal microscopy images, the lamin A/C expression in hMSCs after short-term preconditioning on soft matrices (e.g., So1-St3) is comparable to that in the stiff control (e.g., St1-St3) [Fig. 5(a)]. In comparison, for the long-term preconditioning of hMSCs on soft matrices, lamin A/C expression in So7-St3 group close to that in the soft control (e.g., So7-So3) [Fig. 5(b)]. Quantitative analysis further confirmed that preconditioning on soft matrices for 1 day did not result in significant change of laminA/C expression compared to St1-St3 group, still maintaining relatively high expression level [Fig. 5(c)]. However, extended preconditioning on soft matrices (7 days) led to significant decrease in the expression level of lamin A/C relative to that in St7-St3 group, but comparable to that in So7-So3 group [Fig. 5(d)]. These findings clearly demonstrated that the alteration of nuclear mechanics was closely dependent on the preconditioning time the cells received: short-term mechanical preconditioning had no obvious influence on cell nuclear mechanics, while long-term mechanical preconditioning significantly affects nuclear mechanics of hMSCs. These results indicated that preconditioning duration on soft matrices were also able to impact on the nuclear mechanics of hMSCs in addition to changing the localization of YAP.

**FIG. 6.**
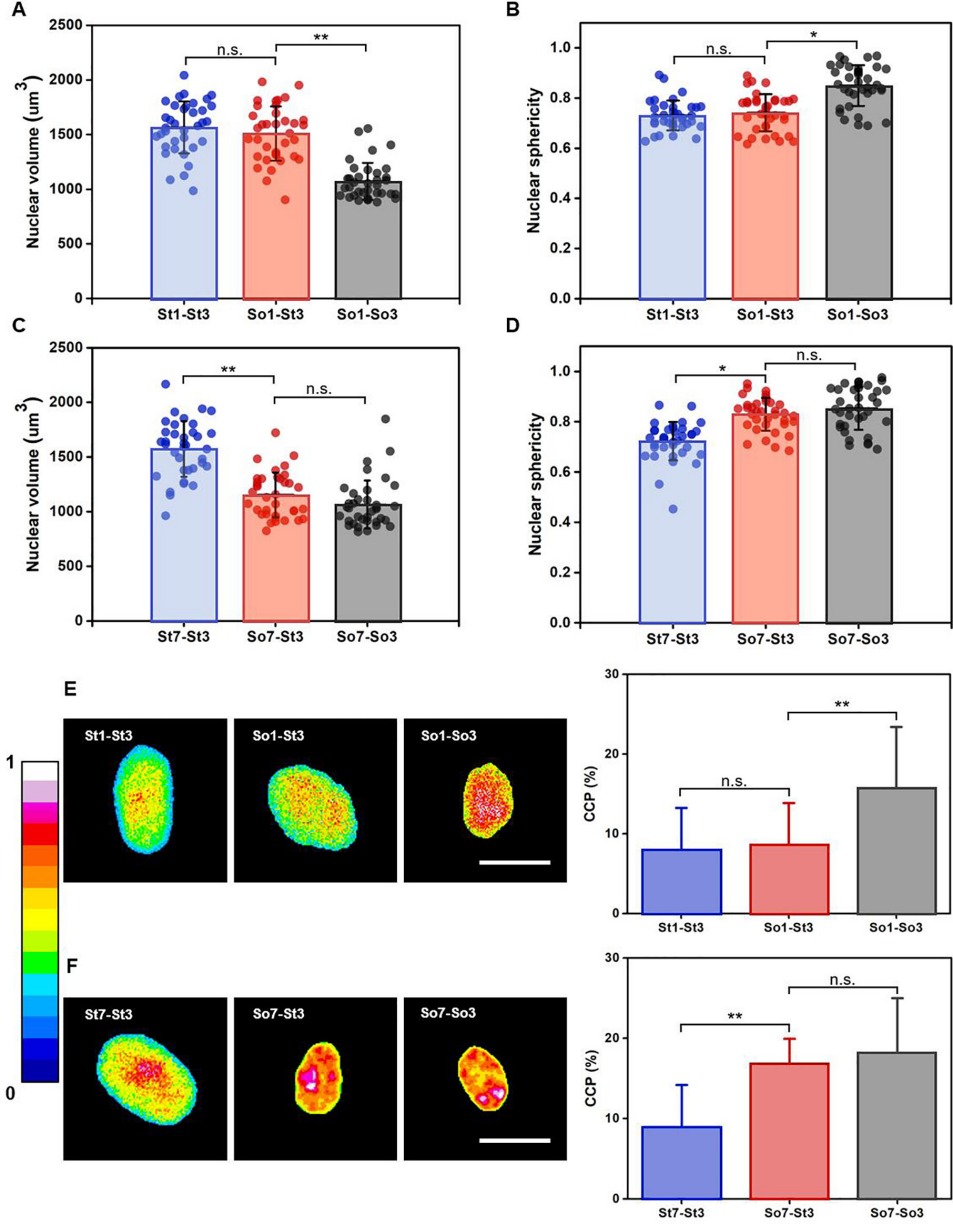
Influences of short-term and long-term mechanical preconditioning on nuclear volume, nuclear shape and chromatin organization of hMSCs. (a) Nuclear volume and (b) nuclear sphericity were quantified (n = 36 cells from biological triplicate) based on nuclear immunostaining with DAPI after short-term preconditioning of hMSCs on soft matrices. ^*^p < 0.05, ^**^p < 0.01. (c) Nuclear volume and (d) nuclear sphericity were quantified (n = 36 cells from biological triplicate) based on nuclear immunostaining with DAPI after long-term preconditioning of hMSCs on soft matrices. ^*^p < 0.05 and ^**^p < 0.01. Chromatin organization and chromatin condensation parameter (CCP) within the cell nuclei of hMSCs receiving (e) short-term and (f) long-term mechanical preconditioning. n = 36 cells from biological triplicate. Scale bar: 20 *μ*m. ^**^p < 0.01.

The nuclear volume and sphericity of the hMSCs after short-term and long-term preconditioning on soft matrices were also assessed [Figs. 6(a)–6(d)]. We observed that the nuclear volume of hMSCs with short-term preconditioning on soft matrices (i.e., So1-St3) was dramatically larger than that in the soft control (i.e., So1-So3), but similar to that in the stiff control (i.e., St1-St3) [Fig. 6(a)]. These indicated a less effect of short-term soft priming on nuclear volume (So1-St3) by maintaining a similar state to those on stiff matrices (St1-St3). A similar trend was also observed in the examination of nuclear sphericity, where 1-day preconditioning of hMSCs on soft matrices (i.e., So1-St3) barely changes the levels of their nuclear sphericity [Fig. 6(b)]. On the contrary, nuclear volume and sphericity of the cells with long-term soft priming (i.e., So7-St3) changed significantly even after transferred to stiff matrices and reached to a similar level to that in the soft control (i.e., So7-So3) [Figs. 6(c) and 6(d)]. This suggested that the hMSCs would memorize the mechanical information of soft matrices during the extended residing and maintain their nuclear volume and sphericity even returned to a stiff microenvironment. Hence, these results revealed that preconditioning duration on soft matrices could effectively regulate nuclear volume and shape.

Considering that nuclear volume and shape can induce alteration in chromatin organization, which may lead to changes in gene expression,^37,38^ we further examined the chromatin organization of hMSCs by staining the nuclei with DAPI. As shown in Figs. 6(e) and 6(f), the nuclei of hMSCs preconditioned on soft matrices for 7 days (i.e., So7-St3) exhibited higher DAPI intensity with much more clusters compared to that preconditioned on soft matrices for 1 day (i.e., So1-St3). Also, the nuclei of hMSCs preconditioned on soft matrices for short term and long term exhibited closer chromatin organization to those in the stiff (i.e., St1-St3) and soft (i.e., So7-So3) controls, respectively. The chromatin condensation parameter (CCP) was further calculated to quantitatively characterize the chromatin condensation within cell nuclei. As presented in Figs. 6(e) and 6(f), CCP values in the So1-St3 group was comparable to that in St1-St3 group. However, CCP values in So7-St3 group were obviously higher than that in St7-St3 group and comparable to that in So7-So3 group. The combined results suggested that the high chromatin condensation regulated by extended preconditioning of hMSCs on soft matrices might be through the mediation of nuclear volume and shape.

### F. Mechanism of extended preconditioning on soft matrices in directing hMSC fate

**FIG. 7.**
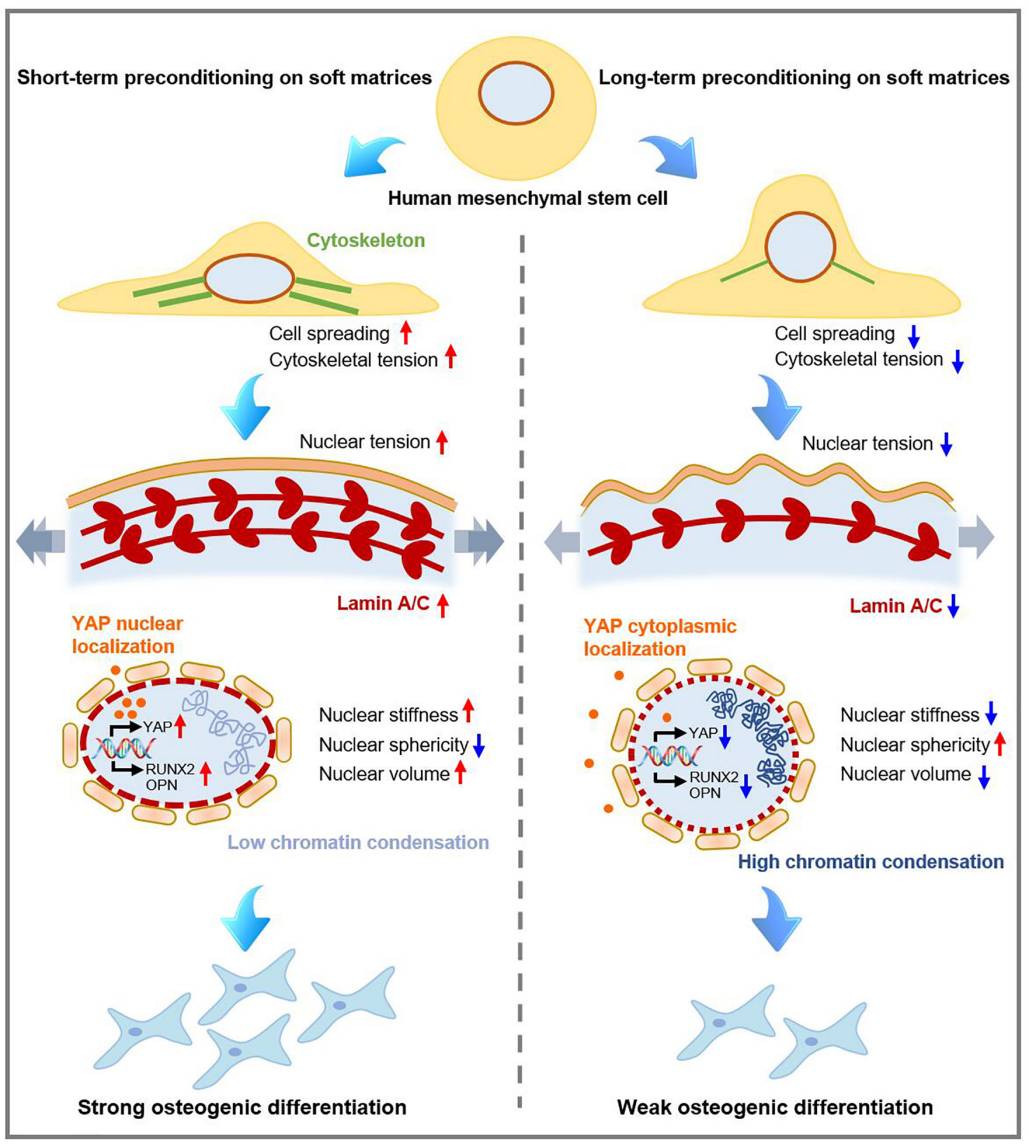
Schematics illustrating the mechanisms of preconditioning duration on soft matrices in directing hMSC fate. Cells receiving short-term or long-term preconditioning on soft matrices spread with different degrees and exhibit differential cytoskeletal tension. Subsequently, cytoskeletal tension-induced subcellular localization of YAP and changes in nuclear features (e.g., nuclear stiffness, sphericity, and volume) that affect chromatin organization jointly guide osteogenic differentiation of hMSCs through regulating transcriptional activity.

Based on the observation above, we propose a hypothesis that extended preconditioning of hMSCs on soft matrices may direct stem cell fate through the regulation of transcriptional activity of YAP and chromatin organization (Fig. 7). Compared to those experienced short-term preconditioning on soft matrices, hMSCs preconditioned on soft matrices for long term exhibited reduced spreading morphology [Figs. 2(b)–2(d)], indicating low cytoskeletal tension. It is noted that cytoskeletal tension is required for the nuclear localization and activation of transcriptional regulator YAP,^39^ regarding as a core mechanotransducer. Thus, long-term preconditioning on soft matrices led to inhibition of YAP transcriptional activity by decreasing the shuttle of YAP from cytoplasm to nucleus [Figs. 4(d) and 4(e)]. This would further suppress the expression of osteogenesis specific proteins (e.g., RUNX2 and OPN) and eventually lead to reduced osteogenic differentiation of hMSCs. In addition, when hMSCs shortly exposed to soft matrices (e.g., So1-St14) were treated with cytochalasin D, about fold decrease in mRNA levels of RUNX2 and OCN compared to that of hMSCs with DMSO treatment (Fig. S9, supplementary material), confirming a crucial role of traction force-mediated mechanotransduction.

Generally, high YAP nuclear localization is in favor of osteogenesis, while it is considered to suppress adipogenesis of MSCs.^40^ Mechanotransduction pathways including focal adhesion formation, cytoskeletal tension generation, and subcellular localization of YAP play an important role. However, some previous studies believed that the adipogenesis signaling pathways were quite different from that of osteogenesis. The subcellular localization of YAP is not the only conclusive factor to guide the adipogenic differentiation of MSCs.^41^ In the current study, hMSCs experiencing long-term and short-term preconditioning on soft matrices exhibited YAP cytoplasmic localization and YAP nuclear localization, respectively, which might be one of main reasons for directing different levels of osteogenic differentiation of hMSCs. Complex cell microenvironment may trigger different signaling pathways to direct cell behavior and fate, and further study is still needed to reveal the underlying mechanobiology mechanism.

Cytoskeletal tension can be transmitted from cytoplasm to the nuclear envelope through the linker of nucleus and cytoskeleton (LINC) complexes.^18,42^ Actomyosin-based cytoskeletal contractility can lead to the generation of nuclear tension. Due to the low cytoskeletal tension present in hMSCs with extended preconditioning on soft matrices, the resultant low nuclear tension is not able to inhibit the phosphorylation and degradation of lamin A/C,^43^ leading to low lamin A/C expression. High lamin A/C expression levels are required for the regulation of serum response factor (SRF) to promote the expression of stress fiber genes, thus further enhancing osteogenic differentiation of MSCs.^17^ Hence, the low lamin A/C expression level in the cells exposed to soft matrices for extended duration could be a critical factor for low osteogenic differentiation of hMSCs in this study.

As previous observed in our study, cell proliferation of hMSCs was differentially affected by soft priming duration [Fig. 2(e)]. However, whether cell proliferation could also affect chromatin organization in cells receiving different preconditioning durations is not clear. To address this, the proliferation of hMSCs receiving short-term and long-term soft priming was both evaluated. After transferring to stiff matrices, the proliferation of hMSCs cultured in osteogenic media was quite slow (Fig. S10, supplementary material). Thus, cell proliferation-related chromatin condensation might be negligible. Alterations in nuclear shape or nuclear volume have suggested to be related to chromatin organization and as a result to modulate gene accessibility.^37,38^ Extended exposure to soft matrices favor the cells to form nuclei with small volume and high degree of sphericity, which contributes to the increase in chromatin condensation. Such high chromatin condensation within the cell nuclei may reduce the gene accessibility required for osteogenic differentiation, thus leading to weakened osteogenic differentiation. hMSCs experiencing short-term preconditioning on soft matrices (e.g., So1-St14) were treated with C646 to induce chromatin condensation, and exhibited obvious decrease in RUNX2 expression compared to the cells treated with DMSO (Fig. S11, supplementary material). These results indicated that extended exposure to soft matrices could reduce osteogenesis capacity of hMSCs by changing chromatin organization. Additionally, the nucleoskeletal protein lamin A/C increases with matrix stiffness and confers nuclear mechanical properties.^17,37^ Previous study has reported that chromatin condensation is one of the major contributors for nuclear mechanics, and high extent of chromatin condensation was accompanied by low nuclear stiffness within the nuclei of MSCs on soft hydrogels with small ligand distance.^44^ This is consistent with what we observed for hMSCs after long-term preconditioning on soft matrices in this work. Such chromatin reorganization associated with nuclear mechanics can further alter the accessibility of chromatin and genes to transcription factors as well as regulate the transcriptional activity, consequently directing the cell fate of hMSCs.

## III. CONCLUSIONS

In summary, we engineered a mechanical culture system to achieve soft priming and subsequent stiff culture of hMSCs for studying time-dependent effect of preconditioning on soft matrices on directing stem cell fate. hMSCs experienced long-term preconditioning on soft matrices retained their past mechanical information, which consequently influenced their osteogenic differentiation even after transferred to stiff matrices. In contract, cells preconditioned on soft matrices for short term exhibited neglect impact from past mechanical information as well as their osteogenesis. The mechanism underlying these findings might be in part by regulating YAP transcriptional activity through changing its subcellular localization. Additionally, chromatin organization could contribute to the hMSC fate determination through the mediation of nuclear mechanics, shape, and volume. This work offers an insight into the understanding of critical effect of time-dependent soft priming on hMSC fate and potential mechanism explaining the phenomena observed in diseased periodontal tissue. The facile approach for regulating cell behavior and fate determination from a mechanobiological perspective also provides a universal platform for other biomedical studies.

## IV. METHODS

### A. Fabrication and characterization of stiff and soft matrices

Polydimethylsiloxane (PDMS) substrates were prepared by using SYLGARD^TM^ 184 silicone elastomer kit (Dow Corning Corporation, USA). The mixtures of base and curing agent were mixed thoroughly and placed into the plastic petri dishes. By changing mass ratio of base to curing agent (e.g., 47:1 and 51:1), stiff and soft PDMS substrates were obtained by incubation of two mixtures at 60 °C for 6 h and cooling at room temperature overnight. PDMS disks with diameter of 8 mm were produced by cutting with biopsy punches. After sterilization, PDMS disks were washed with phosphate buffer saline (PBS) for three times. Collagen type I was extracted from the tendons of rat tail according to previously reported methods.^45^ The prepared lyophilized collagen was weighted and added in 10 mM HCl and gently stirred at 4 °C for 48 h until it was completely dissolved to obtain 5 mg/ml collagen solution. After being neutralized by 2 M NaOH, collagen solution was carefully poured into a petri dish containing clean PDMS disks. Finally, this Petri dish was put into an incubator (5% CO_2_, 37 °C) overnight for collagen coating. For characterization of mechanical property, PDMS substrates before and after collagen modification were compressed (compression rate: 1 mm/min) by a BOSE ELF 3200 dynamic mechanical analyzer (BOSE, USA). The Young's modulus of each PDMS substrate was calculated according to our previously published method.^34^ For topological microstructure characterization, stiff and soft PDMS substrates with collagen modification were freeze-dried and surface-coated through sputter coating with Au for scanning electron microscopy (SEM) imaging. The topological microstructure and pore width of the collagen pattern were observed by a TM4000Plus SEM (Hitachi, Japan) with an accelerating voltage of 15 kV.

### B. Cell culture

hMSCs were isolated from fresh periodontal membranes that were obtained from human teeth following the protocol described in our previous work.^46^ Briefly, healthy human teeth were obtained from three young individuals (12–16 years old) who were treated for orthodontic reasons at the Stomatology Hospital of Xi'an Jiaotong University. In this study, the periodontal membrane surrounding the root was cut and digested in collagenase type I with concentration of 3 mg/ml (Sigma-Aldrich, USA) at 37 °C for 30 min. Subsequently, cell suspensions were filtered with a 40 *μ*m cell strainer (Thermo Fisher Scientific, USA) and cultured with DMEM (Gibco, USA) containing 15% fetal bovine serum (Gibco, USA) and 100 U/ml penicillin streptomycin (Sigma-Aldrich, USA) in the incubator (5% CO_2_, 37 °C). Limiting dilution technique was used to gain single cell-derived cultures, then hMSCs were multiple colony-derived after 3–4 passage. hMSCs with the same passage were used in each experiment. Identification of the hMSCs through a flow cytometer was confirmed in our previous study.^46^ For osteogenic differentiation, the growth media were replaced by the osteogenic media containing 10 mM β-glycerophosphate disodium, 50 μg/ml ascorbic acid, and 100 nM dexamethasone.

### C. Cell behavior characterization

A Live/Dead Viability Kit (Life Technologies, USA) was used to character cell viability of hMSCs after cells were transferred to stiff matrices on day 3. According to the suggested protocol, cells were incubated with 0.05% v/v calcein-AM and 0.2% v/v propidium iodide at 37 °C for 15 min in the dark. Then, cells were washed with PBS and imaged using an FV3000 confocal microscope (Olympus, Japan). Data were analyzed by ImageJ software.

A Cell Count Kit-8 (CCK-8, Dojindo, Japan) was used cell proliferation evaluation. When hMSCs were transferred to stiff matrices and cultured for designed days, the growth media containing 10% v/v CCK-8 were added into the culture media and incubated at 37 °C for 4 h. A microplate reader (BioRad Laboratories, USA) was then used to measure the optical density (OD) of the reaction solution at 450 nm. Three replicates were measured for each group. Meanwhile, a standard curve was obtained. Briefly, a cell counting plate was used to count the number of hMSCs in the prepared cell suspension. Cell concentration gradients were obtained by sequentially diluting the cell suspension with growth media in equal proportion. Five cell concentrations were prepared, and six replicates were in each group. Then, cells were seeded in a 96-well plate and cultured for 2 h to attach to the well. After incubation with 10% v/v CCK-8 at 37 °C for 4 h, OD at 450 nm was measured for manufacturing a standard curve with cell number as abscissa (X-axis) and OD as ordinate (Y-axis). By using this standard curve, cell number of hMSCs cultured for designed days under different conditions was calculated for evaluating cell proliferation.

Cell spreading was assessed after hMSCs were transferred to stiff matrices on day 5. Cells were fixed by 4% paraformaldehyde for 15 min, permeabilized with 0.5% Triton for 10 min, and blocked with 5% bovine serum albumin (BSA) for 30 min. Rhodamine-labeled phalloidin (2 *μ*g/ml, Cytoskeleton, Inc., USA) and 4′,6-diamidine-2′-phenylindole dihydrochloride (DAPI, 1 mg/ml, Sigma, USA) were used at 37 °C for 15 and 10 min for staining F-actin and nuclei, respectively. Images were taken by an FV3000 confocal microscopy (Olympus, Japan), and cell aspect ratio and cell area were analyzed by ImageJ software. 15 cells from biological triplicate were used for the quantitative analysis.

After short-term and long-term exposure to soft matrices, hMSCs were transferred to stiff matrices and cultured subsequently for 5 days. Cells were fixed by 4% paraformaldehyde for 15 min, permeabilized with 0.5% Triton for 10 min, and blocked with 5% BSA for 30 min. Then, cells were incubated with anti-Integrin beta 1 antibody (1:200, Abcam, ab30394) at 4 °C overnight. Goat anti-mouse IgG (Alexa Fluor^®^ 488) (1:1000, Abcam, ab150113) was used as secondary antibodies at 4 °C for 1 h. Cell nuclei were stained with DAPI. An FV3000 confocal microscope (Olympus, Japan) was used for imaging, and ImageJ software was utilized to measure mean fluorescence intensity for quantitation of integrin β1 expression. Biological triplicate with three images per replicate were used for the quantitative analysis.

### D. Cell differentiation characterization

At the early stage of osteogenic differentiation, alkaline phosphatase (ALP) activity was used to evaluate the degree of osteogenic differentiation of hMSCs. On day 10 after cell transfer to stiff matrices, cells were washed with PBS for three times and then stained with a BCIP/NBT alkaline phosphatase color development kit (C3206, Beyotime Institute of Biotechnology) at room temperature for 20 min. Images were taken through an ECLIPSE Ti inverted microscope (Nikon, Germany). To quantify ALP expression, at designed time points, cells were collected and lysed by RIPA lysis buffer (Thermo Fisher Scientific, USA) on ice for 20 min. Then, the lysates were centrifuged at 10 000 rpm for 12 min at 4 °C and analyzed by an ALP Assay Kit (A059–2–2, Nanjing Jiancheng Bioengineering Institute, China) according to the manufacturer's instructions. The ALP activity was normalized by the content of total protein determined by an Enhanced BCA Protein Assay Kit (P0010, Beyotime, China). ALP was performed in biological triplicate.

At the middle stage of osteogenic differentiation, expression of osteogenesis specific proteins, such as RUNX2 and osteopontin (OPN), was assessed. On day 14 after cell transfer to stiff matrices, cells were washed with PBS for three times initially. Then, cells were fixed by 4% paraformaldehyde for 15 min, permeabilized with 0.5% Triton for 10 min, and blocked with 5% BSA for 30 min. Next, cells were incubated with anti-RUNX2 antibody (1:100, Abcam, ab76956) and anti-OPN antibody (1:100, Abcam, ab8448) at 4 °C overnight, respectively. Goat anti-mouse IgG (Alexa Fluor^®^ 488) (1:1000, Abcam, ab150113) and goat anti-rabbit IgG (Alexa Fluor^®^ 488) (1:1000, Abcam, ab150077) were used as secondary antibodies at 4 °C for 1 h. Cell nuclei were stained with DAPI. To determine the role of chromatin organization in soft priming-regulated osteogenesis capacity, hMSCs with short-term preconditioning on soft matrices (e.g., 1 day of exposure) were treated with C646 (10 *μ*M, MedChemExpress, HY-13823) according to the manufacturer's protocols. Cells with DMSO treatment were controls. RUNX2 expression in hMSCs (e.g., So1-St14) was evaluated on day 14. An FV3000 confocal microscope (Olympus, Japan) was used for imaging, and ImageJ software was utilized to measure mean fluorescence intensity for quantitation of protein expression. Biological triplicate with 3 images per replicate were used for the quantitative analysis.

The mRNA levels of RUNX2 and OPN were evaluated for the hMSCs cultured in osteogenic media for 14 days with varied preconditioning time on soft matrices. RNA Isolation Kit (QIAGEN, Germany) was initially used to isolate the total RNA of hMSCs, and PrimeScript RT Reagent Kit with genomic DNA Eraser (TaKaRa Bio, China) was then used to synthesize the complementary DNAs. Then the complementary DNAs were mixed with the SYBR Premix Ex Taq II Kit (TaKaRa Bio, China) and loaded in a 7500 Fast Real-Time PCR System (Applied Biosystems, CA) for 40 cycles at 95 °C for 3 s and 60 °C for 30 s. GAPDH was served as a housekeeping gene and the forward and reverse primers of GAPDH, RUNX2, and OPN are shown in Table I. Three independent RNA preparations were measured for each group. The RUNX2 and OPN messenger RNA expression values were calculated by the 2^–*ΔΔ*Ct^ method (e.g., *Δ*Ct = the mean cycle threshold Ct of the target gene—the mean Ct of GAPDH; *ΔΔ*Ct = *Δ*Ct of experimental group—*Δ*Ct of control group). Cells in St14 group were set as controls, and cells in So1-St14, So4-St14, and So7-St14 groups were set as experimental groups for gene expression. To determine the role of traction force-mediated mechanotransduction, hMSCs shortly exposed to soft matrices (e.g., 1 day of exposure) were treated with cytochalasin D (Maokangbio, MZ5802) according to the manufacturer's protocols. Cells treated with DMSO were set as controls.

**TABLE I.** Sequence of the forward and reverse primers for RT-PCR.

Genes	Forward primers	Reverse primers
RUNX2	CACAGAGCAATTAAAGTTAC	CTAGGTTTAGAGTCATCAAG
OPN	GTGCAGAGTCCAGCAAAGGT	TCAGCCAACTCGTCACAGTC
GAPDH	GGACCTGACCTGCCGTCTAG	TAGCCCAGGATGCCCTTGAG

At the late stage of osteogenic differentiation, alizarin red staining was used to determine the production of mineralized nodules. On day 21 after cell transfer to stiff matrices, cells were washed with PBS, fixed by 4% paraformaldehyde for 15 min, and stained with 40 mM alizarin red S (Sigma-Aldrich, USA) solution at 37 °C for 15 min. For quantitative result of alizarin red staining, the stained cells were incubated in disodium hydrogen phosphate solution containing cetylpyridinium chloride (10% w/v, 500 *μ*l) at 37 °C for 15 min. A microplate reader (BioRad Laboratories, USA) was used to measure optical density of reaction solution at 570 nm. Alizarin red staining was performed in biological triplicate.

### E. Immunostaining of YAP and lamin A/C

After cell receiving short-term and long-term preconditioning on soft matrices, cells were transferred to stiff matrices and cultured subsequently for designed times. cells were fixed by 4% paraformaldehyde for 15 min, permeabilized with 0.5% Triton for 10 min, and blocked with 5% BSA for 30 min. To analyze subcellular localization of YAP, anti-YAP (1:200, Abcam, ab52771) primary antibody was added and incubated with cells at 4 °C overnight. After removing this primary antibody, cells were incubated with goat anti-rabbit IgG (Alexa Fluor^®^ 488) (1:1000, Abcam, ab150077) regarding as secondary antibody, rhodamine-labeled phalloidin (2 *μ*g/ml, Cytoskeleton, Inc., USA), and DAPI (1 mg/ml, Sigma, USA) at 4 °C for 1 h. After washing with PBS twice, cells were imaged by an FV3000 confocal microscope (Olympus, Japan). By measuring the mean fluorescence intensity of the YAP staining in the nucleus and cytoplasm by ImageJ, YAP Nuc/Cyt ratio was calculated by dividing the mean fluorescence intensity of the YAP staining in the nucleus by that in the cytoplasm. To determine nuclear mechanics, anti-LaminA + Lamin C (1:200, Abcam, ab108595) primary antibody was added and incubated with cells at 4 °C overnight. Then, cells were incubated with goat anti-rabbit IgG (Alexa Fluor^®^ 488) (1:1000, Abcam, ab150077) regarding as secondary antibody and DAPI (1 mg/ml, Sigma, USA) at 4 °C for 1 h. After washing with PBS twice, cell nuclei were imaged by an FV3000 confocal microscope (Olympus, Japan). Expression of lamin A/C was quantified by measuring the mean fluorescence intensity of lamin A/C staining in the nucleus by ImageJ.^44^ 15 cells from biological triplicate were used for quantitative analysis.

### F. Analysis of nuclear volume and sphericity

Nuclear volume was confirmed from DAPI-stained cells that were imaged (z-interval: 0.5 *μ*m, magnification: 100 ×, and resolution: 9.65 pixels per micrometer) by an FV3000 confocal microscope (Olympus, Japan) and was calculated from confocal laser scanning microscopy (CLSM) image z-stacks by using a custom-designed MATLAB routine.^47^ Image denoising based on the binary thresholding function in MATLAB was performed on dozens of images from each analyzed focal plane. By using the Canny Edge Detector operator, the boundary of the cell nucleus was determined in each image. To obtain the cross-sectional area of each slice, the edge was dilated and refilled. Finally, by multiplying the average area from two adjacent slices by the z-interval and integrating all values, the nuclear volume was measured. Nuclear sphericity was calculated by dividing the surface area of a perfect sphere with the same volume by the measured surface area of the nucleus. 36 cells from biological triplicate were used for quantitative analysis.

### G. Analysis of chromatin organization

Chromatin organization within the cell nuclei of hMSCs under different conditions was characterized by creating a heatmap of the DAPI intensity and calculating the chromatin condensation parameter (CCP). Initially, an FV3000 confocal microscope (Olympus, Japan) was used to take the 2D fluorescence image of individual nucleus stained by DAPI. Then, this image was converted to grayscale. By using the function of image-lookup table-spectrum in ImageJ software, a heatmap of the nucleus was obtained. In addition, a 2D fluorescence image of nucleus was used with a MATLAB code for calculating the CCP, which was described in detail previously. Briefly, fluorescence images should be saved as 8 bit .tif files in RGB values. Each image can only contain one cell nuclei on a black background. All images must be placed in the working directory with the MATLAB file. The code will cycle through all images in folder. Chromatin condensation analysis was performed by using the sobel edge finding method. Image analysis step output, input variables, results output setup, and core algorithm were referred in a previous work.^48^ 36 cells from biological triplicate were used for quantitative analysis.

### H. Statistical analysis

Characterization of PDMS substrates, for instance stiffness, was performed in n = 3 (with biological replicates); Cell proliferation, ALP, alizarin red staining, and PCR were performed in biological triplicate; Image-based quantifications were performed in at least biological triplicate and detailed throughout the methods and figure legends. Analyses of cell area, cell aspect ratio, nuclear surface area, fluorescence intensity, and YAP Nuc/Cyt ratio were performed by using ImageJ. Nuclear volume and chromatin organization were quantified by using a custom MATLAB script. Statistical analyses in the current study were performed by GraphPad Prism 7. Meanwhile, this software was used to perform t tests to determine significance. All data were reported as means ± standard deviation (S.D). Significance levels were set at ^*^p < 0.05 and ^**^p < 0.01.

## SUPPLEMENTARY MATERIAL

See the supplementary material for details including Figs. S1–S11 in this study.

## Data Availability

The data that support the findings of this study are available from the corresponding author upon reasonable request.
